# Preparation and Electrochemical Properties of Porous Carbon Nanofiber Electrodes Derived from New Precursor Polymer: 6FDA-TFMB

**DOI:** 10.3390/polym12081851

**Published:** 2020-08-18

**Authors:** Byeongil Jeon, Taehwa Ha, Dong Yun Lee, Myung-Seok Choi, Seung Woo Lee, Kyung-Hye Jung

**Affiliations:** 1 School of Advanced Materials and Chemical Engineering, Daegu Catholic University, Gyeongsan 38430, Korea; qzpqzp12@gmail.com; 2 School of Chemical Engineering, Yeungnam Universiry, Gyeonsan 38430, Korea; lonu7324@gmail.com; 3 Department of Polymer Science and Engineering, Kyungpook National University, Daegu 41566, Korea; dongyunlee@knu.ac.kr; 4 Division of Chemical Engineering, Konkuk University, Seoul 143701, Korea; mchoi@konkuk.ac.kr

**Keywords:** carbon nanofiber electrodes, EDLCs, 6FDA-TFMB, electrospinning, porous carbon

## Abstract

Porous carbon nanofibers (CNFs) with high energy storage performance were fabricated with a single precursor polymer, 6FDA-TFMB, without the use of any pore-generating materials. 6FDA-TFMB was synthesized, electrospun, and thermally treated to produce binder-free CNF electrodes for electrochemical double-layer capacitors (EDLCs). Highly porous CNFs with a surface area of 2213 m^2^ g^−1^ were prepared by steam-activation. CNFs derived from 6FDA-TFMB showed rectangular cyclic voltammograms with a specific capacitance of 292.3 F g^−1^ at 10 mV s^−1^. It was also seen that CNFs exhibit a maximum energy density of 13.1 Wh kg^−1^ at 0.5 A g^−1^ and power density of 1.7 kW kg^−1^ at 5 A g^−1^, which is significantly higher than those from the common precursor polymer, polyacrylonitrile (PAN).

## 1. Introduction

Electrochemical double-layer capacitors (EDLCs) store charge using ion adsorption at the electrode surface without redox reactions, and thus have high power and long cyclability. The development of nanostructured carbon electrode materials such as carbon nanotubes (CNTs) or graphene enables high EDLC performance due to a large number of active sites on their surface [1,2].

Carbon nanofibers (CNFs) are a good candidate for EDLC electrode materials due to their large surface area-to-volume ratio, reliable electrical conductivity, and well-controlled nanostructures. They are generally fabricated by thermal treatment of electrospun precursor nanofibers such as polyacrylonitrile (PAN), polybenzimidazole (PBI) [3], lignin [4], and cellulose [5]. Among precursors, aromatic polyimides are good precursor candidates due to their high thermal stability and well-defined molecular structure, and they have been mainly investigated as carbon molecular sieve membranes for gas separation [6,7,8,9,10,11]. The molecular structure of polyimides can be controlled by the selection of dianhydride and diamine, which is crucial to determine carbonization behavior. Additionally, they can be converted to porous carbon due to the release of nitrogen caused by the decomposition of imide rings during carbonization (around 800 °C) [12].

Polyimides containing hexafluoroisopropylidene diphthalic anhydride (6FDA) moiety show high thermal stability and free volume, which makes them outstanding candidates for CNF precursors [13,14,15]. Among them, a polyimide consisting of 6FDA and 2-2′-bis(trifluoromethyl)benzidine (TFMB) contains four trifluoromethyl (CF_3_–) groups in the repeating unit, and the decomposition of CF_3_– groups can additionally generate pores during pyrolysis [16]. In addition, high free volume caused by CF_3_– groups is favorable for polymer chain rearrangement during thermal treatment. It has also been reported that porous CNFs can be prepared when the precursor polymers have high free volume [17,18,19]. Therefore, 6FDA-TFMB seems likely to produce porous CNFs without using pore-generating materials.

Here we report synthesis of CNFs via pyrolysis of electrospun 6FDA-TFMB nanofibers, which serve as binderless electrodes in EDLCs. The surface area and porosity of CNFs were investigated by nitrogen adsorption/desorption measurements while the carbon microstructures were investigated by Raman spectroscopy. The electrochemical properties were studied by assembling coin cells with an aqueous electrolyte.

## 2. Materials and Methods

The synthesis of 6FDA-TFMB was carried out according to literature procedures [20,21]. The solution of an equivalent mol of 6FDA-TFMB in isoquinoline and 1-methyl-2-pyrrolidinone (NMP) was heated at 70 °C for 2 h, refluxed for 5 h, and then precipitated in methanol. The number average molecular weight (*M*_n_) of 6FDA-TFMB was 54,000 with a polydispersity index (PDI) of 1.43 from a gel permeation chromatography (GPC) system using a PL-GPC 210 (Polymer laboratories, Church Stretton, UK).

6FDA-TFMB dissolved in NMP with concentrations of 30–33 wt % was electrospun under applied voltages of 9–15 kV, stabilized at 300 °C under air flow for 3 h, and carbonized at 800 °C under N_2_ for 30 min. To enhance the surface properties, carbonized 6FDA-TFMB was subsequently steam-activated at 700 °C for 30 min with the feed rate of 0.05 mL h^−1^, and annealed at 800 °C for 30 min to remove undesired oxygen-containing functional groups induced by the steam treatment. The CNFs derived from PAN were also prepared for comparison.

Fourier-transform infrared (FTIR) spectroscopy was recorded using a Nicolet 6700 (Thermo Scientific, Waltham, MA, USA) at a 4 cm^−1^ resolution using a liquid-nitrogen-cooled mercury cadmium telluride (MCT) detector. Thermogravimetric analysis (TGA) was carried out with a TGA7 (Perkin-Elmer, Waltham, MA, USA) by heating the 6FDA-TFMB up to 800 °C at a heating rate of 10 °C min^−1^ under nitrogen. Scanning electron microscopy (SEM, SU8220, Hitachi, Japan) was measured to investigate the surface morphology of the precursor and carbon nanofibers. Carbon microstructures were observed with a Raman spectrometer (inVia reflex, Renishaw, Wotton-under-Edge, UK) with a 780 nm laser. The surface and pore properties of the activated CNFs were characterized using N_2_ adsorption/desorption isotherms measured on a Quantachrome (Autosorb-iQ and Quadrasorb SI, Quantachrome, Boynton Beach, FL, USA).

The activated CNFs were directly used as electrodes in the symmetric coin-type cells with two electrodes. Glass fiber separators and 6.0 M of potassium hydroxide were employed as a separator and an electrolyte, respectively. The electrochemical properties were investigated using a WBCS3000S (Wonatech, Seoul, South Korea). From cyclic voltammetry (CV), the specific capacitance (*C*_sp_, F g^−1^) for symmetric cells was calculated using Equation (1) as follows:

(1) $$C_{\mathrm{sp}}=\frac{4}{m}\frac{I}{v}$$

where *m* is the mass of both electrodes (g), *I* the discharge current (A), and *ν* the scan rate (V s^−1^). CV measurements were conducted in the voltage range of −0.5 to 0.5 V with the scan rate range of 10 to 100 mV/s. Cyclic performance was studied by measuring capacitance retention after 5000 cycles under the voltage range of −0.5 to 0.5 V and a scan rate of 100 mV/s.

The galvanostatic charge–discharge test was carried out with a voltage range of 1.0 to 0 V and a discharge current density range of 0.5 to 5 A/g. From the discharge curves, energy density (*E*, Wh kg^−1^) and power density (*P*, W kg^−1^) were calculated using Equations (2) and (3) as follows:

(2) $$E=\frac{I_{d}t_{d}V}{2m}$$

(3) $$P=\frac{t_{d}}{E}$$

respectively, where *I*_d_ is the discharge current (A), *t*_d_ the discharge time (s), and *V* the voltage window (V). Electrochemical impedance spectra (EIS) were evaluated over the frequency range of 100 kHz and 0.1 Hz using a 0.01 V amplitude on a PEC-L01, Peccek, Japan.

## 3. Results and Discussion

**Figure 1:**
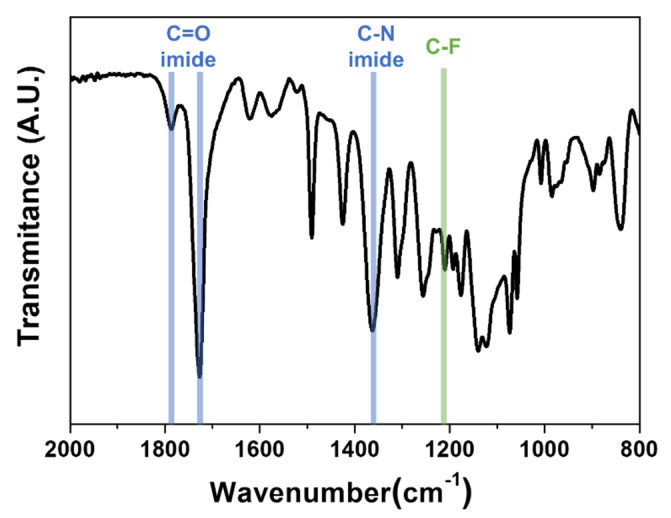
FTIR spectrum of synthesized 6FDA-TFMB.

The chemical structures of synthesized 6FDA-TFMB were observed using FTIR spectroscopy, as shown in Figure 1. It shows distinctive bands at 1727 and 1787 cm^−1^ for C=O symmetric and asymmetric stretch, and 1362 cm^−1^ for C–N stretch of imide. A peak at 1211 cm^−1^ for C–F stretch is also found.

**Figure 2:**
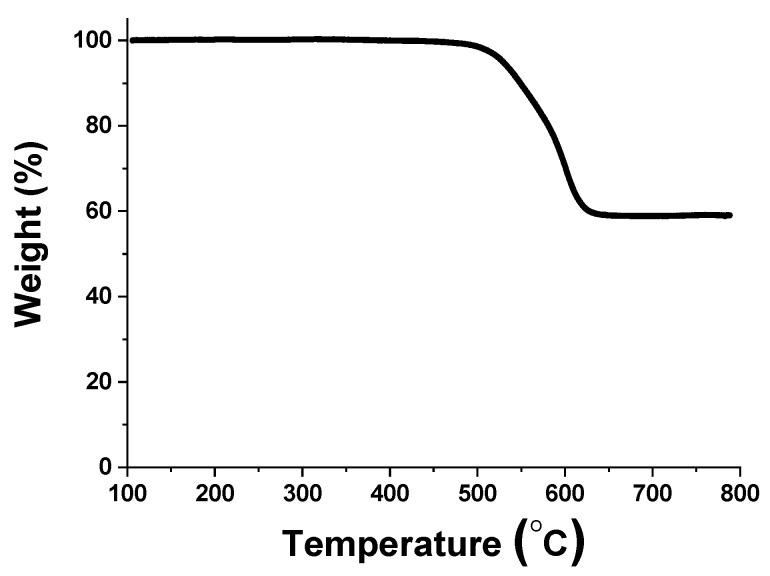
Thermogravimetric analysis of 6FDA-TFMB.

The thermal stability of synthesized 6FDA-TFMB was studied by measuring TGA, as shown in Figure 2, and 6FDA-TFMB seems thermally stable up to 500 °C. It was reported that a breakage of carbonyl groups in the imide ring takes place around 500 °C while benzene ring cleavage was barely found during the pyrolysis of polyimides [12,22,23]. It is also seen that weight loss is less than 50% up to 800 °C.

**Figure 3:**
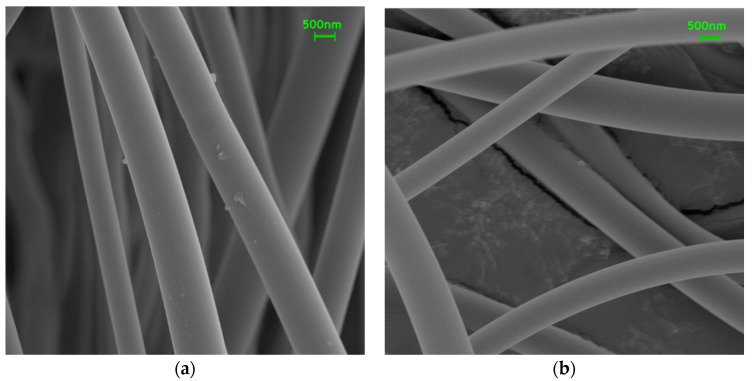
Surface images of (**a**) electrospun and (**b**) carbonized 6FDA-TFMB nanofibers.

6FDA-TFMB was dissolved in NMP and electrospun to produce precursor nanofibers, and their surface image was shown in Figure 3a. The fiber diameter of the 6FDA-TFMB nanofibers is in the range of 660 ± 40 nm. After the pyrolysis process of stabilization at 300 °C and carbonization at 800 °C, the nanofibrous structure successfully remains with a reduced diameter of 630 ± 30 nm, as shown in Figure 3b.

**Figure 4:**
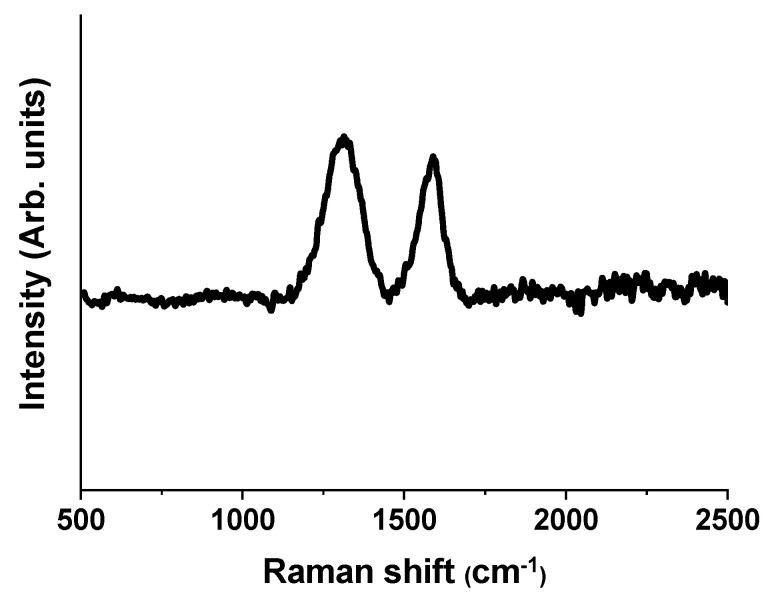
Raman spectroscopy of carbon nanofibers (CNFs) derived from 6FDA-TFMB.

The microstructures of carbon derived from 6FDA-TFMB were investigated via Raman spectroscopy. In Figure 4, two sharp peaks appear near 1595 and 1310 cm^−1^, associated with E_2_g_2_ graphitic crystallites (G-band) and structure defects and disordered carbon (D-band), respectively. It is also seen that the ratio of peak intensities, *I*_D_/*I*_G_ is 1.8, indicating the conversion of 6FDA-TFMB to predominantly disordered carbon via pyrolysis with a typical degree of graphitization for carbon derived from precursor polymers.

CNFs have been mainly produced via either catalytic chemical vapor deposition (CVD) growth or electrospinning followed by thermal treatment [24,25]. Compared to CVD grown ones, CNFs prepared via electrospinning have relatively high contents of amorphous carbon, which may cause lower electrical conductivity. Still, it is advantageous to use electrospun nanofibers for CNF synthesis since they can be readily fabricated, and they have large surface area and porosity.

When a voltage is applied in EDLCs, potential-driven accumulation of ions takes place to counterbalance the surface charge of electrodes, which is the double layers formed at the electrode/electrolyte interface [26]. Due to the energy storage mechanism of EDLCs, many research studies focus on improving the surface porosity of CNFs using sacrificial porogens [27,28].

**Figure 5:**
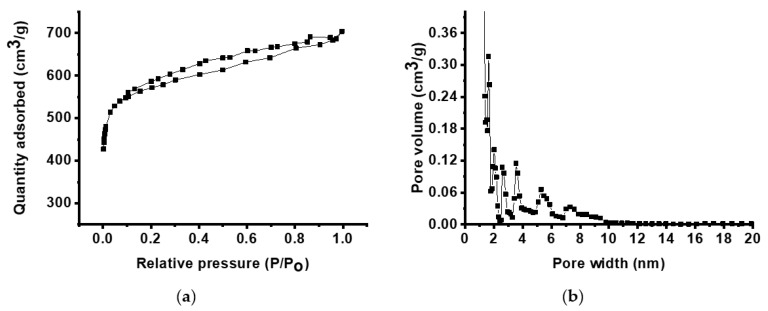
Nitrogen adsorption/desorption isotherms (**a**) and pore size distribution (**b**) of CNFs derived from 6FDA-TFMB.

The CNFs were steam-activated to achieve high surface properties, and the specific surface area and pore sizes were calculated using the Brunauer–Emmett–Teller (BET) method and density functional theory (DFT), respectively. Table 1 shows the summarized nitrogen sorption/desorption data, based on Figure 5. The activated CNFs derived from 6FDA-TFMB show a high surface area of 2210 m^2^ g^−1^ without using porogens. In addition, it is seen that 28% of the pores are meso-sized, which can facilitate the accessibility of electrolyte ions.

6FDA-based polyimides show high thermal stability due to the benzene rings in their backbone and high flexibility due to the bulky fluorinated substituents, which is important for carbon precursors. Polymer chain flexibility is critical for carbon precursors as the high mobility of polymer chains is favorable for polymer chain rearrangement during the stabilization process, and high free volume can be translated to high porosity in carbon [17,18,19]. Previously, 6FDA-durene (2,3,5,6-tetramethyl-1,4-phenylenediamine) was employed as a single CNF precursor, and highly porous CNFs with a surface area of 1210 m^2^ g^−1^ and pore volume of 0.580 cm^3^ g^−1^ were successfully prepared [17]. 6FDA-TFMB exhibits a lower glass transition temperature (335 °C) as it contains four CF_3_– groups than 6FDA-durene (424 °C) containing two CF_3_– groups [29,30].

Additionally, Ohta et al. reported that carbon films prepared by carbonization of 6FDA-TFMB above 600 °C show higher surface area and pore volume than those from polyimides containing less CF_3_– groups as a result of the additional pore generation caused by the evolution of gaseous fluorine compounds such as CF_3_, CF, CH_2_F_2_ and COF_2_ [16]. Therefore, it can be concluded that high free volume and CF_3_– groups of 6FDA-TFMB enables the production of highly porous carbon.

For electrochemical testing, symmetric coin cells were assembled with two identical electrodes of activated CNFs and an aqueous electrolyte, KOH. EDLC performances were studied via CV and galvanostatic charge–discharge testing. The electrochemical properties of CNFs derived from 6FDA-TFMB and PAN are summarized in Table 2.

**Figure 6:**
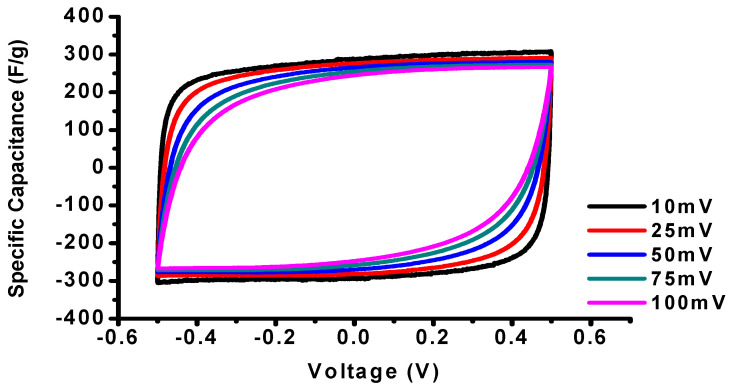
Cyclic voltammograms (CVs) of CNF electrodes derived from 6FDA-TFMB.

In Figure 6, CNF electrodes derived from 6FDA-TFMB show rectangular CVs without faradaic peaks with a high specific capacitance of 292.3 F g^−1^ at 10 mV s^−1^. As the scan rate increases from 10 to 100 mV s^−1^, the specific capacitance is decreased from 292.3 to 246.1 F g^−1^ due to the limited interaction between the electrolyte ions and the electrode surface.

**Figure 7:**
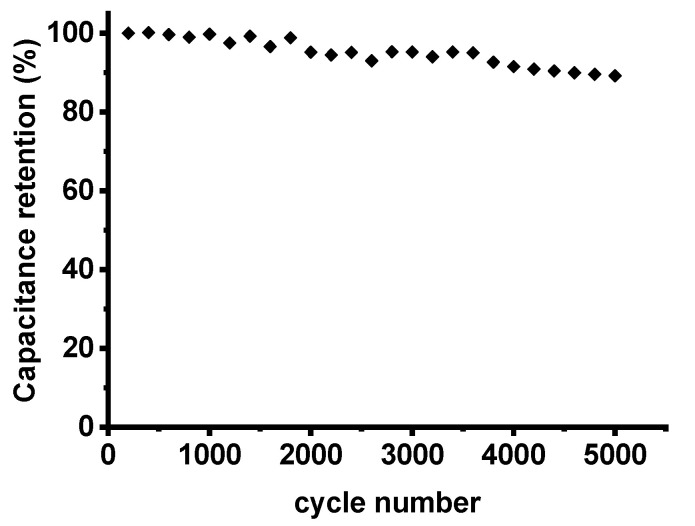
Cyclic performance of CNF electrodes derived from 6FDA-TFMB.

To evaluate cycling stability, CVs were measured for 5000 cycles and the specific capacitance at 100 mV s^−1^ for every 100 cycles is shown in Figure 7. CNF electrodes derived from 6FDA-TFMB exhibit a capacitance retention of 89.2% after 5000 cycles, which is comparable to those from PAN. 

**Figure 8:**
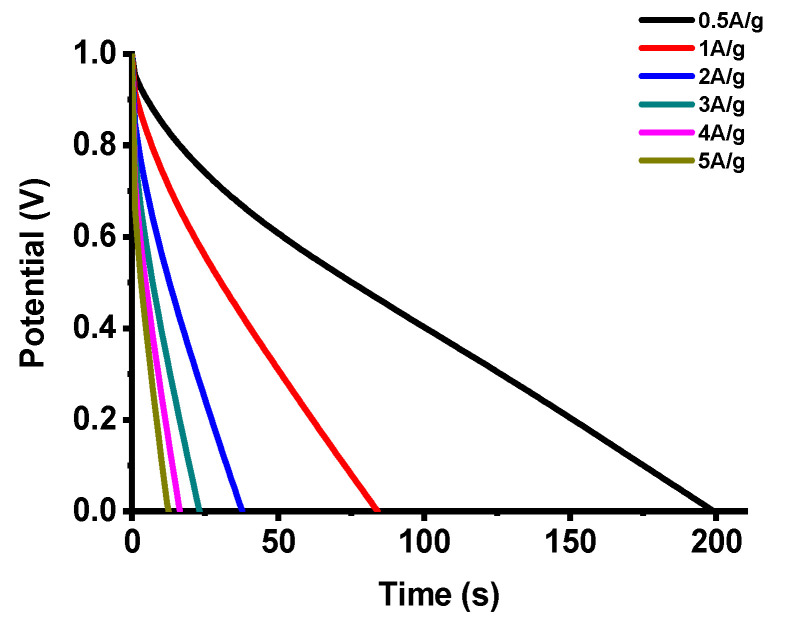
Discharge curves of CNF electrodes derived from 6FDA-TFMB.

The energy and power densities in Table 2 were calculated from galvanostatic discharge times and ohmic drops in discharge curves over a discharge current density range of 0.5 to 5 A g^−1^, as shown in Figure 8. The activated CNFs derived from 6FDA-TFMB show a significantly higher energy density of 13.1 Wh kg^−1^ at 0.5 A g^−1^ than those from PAN due to the long discharge time resulting from high surface area and porosity.

The breakdown voltage for supercapacitors is mainly determined by the type of electrolyte. For example, the maximum voltage for supercapacitors with aqueous electrolytes, such as KOH, in this study is 1.1 V. For electrode materials, the surface properties are critical to widen the operating of the electrolyte. CNF electrodes derived from 6FDA-TFMB exhibit high surface area and porosity, and cause low IR drops and high energy densities, as shown in Figure 8. 

Electrochemical properties of CNFs have been typically improved by adding pore-generating materials to precursor polymers such as PAN. Recently, poly(acrylic acid) (PAA), poly(ethylene glycol) (PEG), and poly(methyl methacrylate) (PMMA) have been utilized as porogens to produce porous CNFs, and energy storage performances were successfully improved compared to CNFs derived from PAN only [31,32,33]. Porous CNF electrodes derived from a single precursor such as 6FDA-durene were also reported [17,34]. The higher surface area and porosity of CNFs derived from 6FDA-TFMB result in higher electrochemical properties compared to those from 6FDA-durene. 

**Figure 9:**
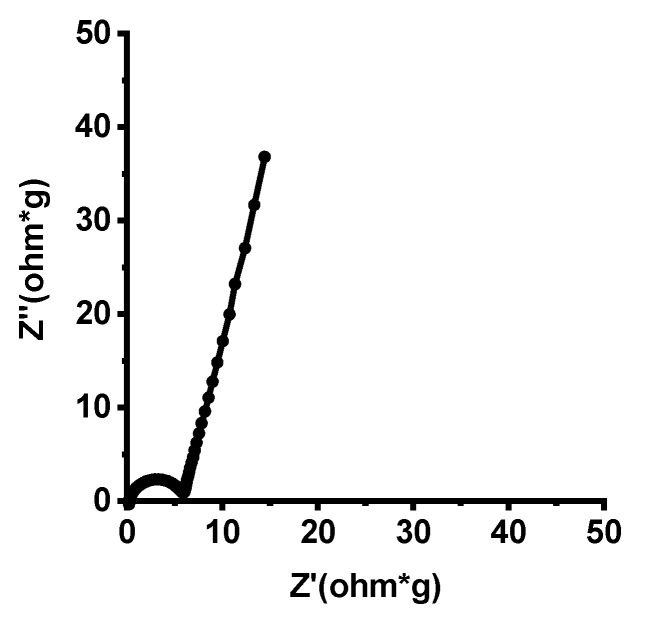
Electrochemical impedance spectra (EIS) of CNF electrodes derived from CNF electrodes derived from 6FDA-TFMB.

The EIS measurement was carried out as shown in Figure 9. The intercept between the semicircle and the real axis in the high frequency region represents the sum of the resistance of the electrolyte, the intrinsic resistance of the electrode, and the interfacial resistance at electrode/current collectors. The charge-transfer resistance (Rct) of the electrodes, the half of the value of the real semicircle in the high frequency region, is 2.95 Ω cm^−1^, which is drastically lower than those from PAN (8.50 Ω cm^−1^). The resistance of electrode materials determines the size of the semicircle at high frequency while electrochemical double-layer formation at the electrode surface determines the slope of the plots at low frequency [35]. Highly porous CNF electrodes enable fast electrolyte ion accumulation and diffusion onto the electrode surface, resulting in low resistance. It also causes significantly higher energy storage performances compared to CNFs derived from PAN or other precursors. 

Compared to PAN, the cost of 6FDA-TFMB might be high. However, PAN has high crystallinity caused by high interaction between nitrile groups, which causes poor processability, and PAN-based CNFs have low electrochemical performances for electrode materials. To solve this, copolymerization or blends of PAN have been used, which can increase the production cost and cause lower mechanical stability. Regarding the electrochemical performance, we have not found any drawbacks of using 6FDA-TFMB so far. We agree that more studies are required to determine the possible drawbacks of using 6FDA-TFMB as electrode materials for supercapacitors.

Recently, carbon composite electrodes using transition metal oxides or conducting polymers have been widely investigated to improve electrochemical properties such as energy density caused by pseudocapacitance [36,37]. It has been reported that pseudocapacitor electrodes were prepared by applying a surface coating of MnO_2_ onto the CNFs, which resulted in high energy storage performances [38,39]. Additionally, CNF electrodes have been prepared by thermal treatment of PAN and polyvinylpyrrolidone (PVP) to increase nitrogen content for high electrochemical properties [40,41]. It seems reasonable that these strategies can be applied to CNFs derived from 6FDA-TFMB to obtain even better energy storage performances by providing highly porous carbon support.

## 4. Conclusions

Porous CNF electrodes were prepared by pyrolysis of 6FDA-TFMB without the addition of porogens due to four CF_3_– groups and high free volume of the precursor polymer. Successful conversion of 6FDA-TFMB to carbon was confirmed via Raman spectroscopy with an *I*_D_/*I*_G_ of 1.8. Surface properties were further improved by activating CNFs with steam, and the resultant CNF electrodes exhibited a high surface area of 2210 m^2^ g^−1^ and pore volume of 1.055 cm^3^ g^−1^ with 28% of meso-sized pores. Symmetric coin cells were assembled with binder-free CNF electrodes and a specific capacitance of 292.3 F g^−1^ (at 10 mV s^−1^), maximum energy density of 13.1 Wh kg^−1^ (at 0.5 A g^−1^) and power density of 1.7 kW kg^−1^ (at 5 A g^−1^) were obtained, which is significantly higher than those from PAN. It can be concluded that the high surface area and porosity of CNFs derived from 6FDA-TFMB enables exceptional EDLC performance.

## Figures and Tables

**Table 1:** Surface properties of CNFs derived from 6FDA-TFMB.

SSA ^1^ (m^2^ g^−1^)	TPV ^2^ (cm^3^ g^−^^1^)	*V*_micro_^3^ (cm^3^ g^−1^)	*V*_meso_^4^ (cm^3^ g^−1^)
2210	1.055	0.757	0.298

^1^ SSA: specific surface area. ^2^ TPV: total pore volume. ^3^ *V*_micro_: micro-pore (<2 nm) volume. ^4^ *V*_meso_: meso-pore (2–50 nm) volume.

**Table 2:** Electrochemical performances of CNF electrodes derived from 6FDA-TFMB and polyacrylonitrile (PAN).

Precursor	6FDA-TFMB	PAN
Specific capacitance (F g^−1^) at 10 mV s^−1^	292.3	210.7
Energy density (Wh kg^−1^) at 0.5 A g^−1^	13.1	8.0
Power density (kW kg^−1^) at 5 A g^−1^	1.7	1.7
